# Open and Periodic Boundary Conditions in Statistical Mechanics: A Case Study of the Antiferromagnetic Ising Chain

**DOI:** 10.3390/e28070727

**Published:** 2026-06-24

**Authors:** Katarína Karl’ová, Jozef Strečka

**Affiliations:** Department of Theoretical Physics and Astrophysics, Faculty of Science, Pavol Jozef Šafárik University in Košice, Park Angelinum 9, 040 01 Košice, Slovakia; katarina.karlova@upjs.sk

**Keywords:** Ising chain, transfer matrix, open and periodic boundary conditions, magnetization plateaus, magnetic susceptibility

## Abstract

The transfer-matrix method is employed to investigate a spin-1/2 Ising chain under open and periodic boundary conditions. It is demonstrated that finite-size Ising chains with antiferromagnetic coupling may exhibit significantly distinct magnetic behavior under open and periodic boundary conditions. While the open Ising chains display intriguing magnetic features regardless of the system size, mainly due to a specific contribution of boundary spins, the magnetic behavior of closed Ising chains depends basically on the number of spins. The closed Ising chains with an odd number of spins are subject to a geometric spin frustration leading to an additional plateau in the magnetization curve, which is naturally absent in the closed Ising chains with an even number of spins. Despite different microscopic origins, the magnetization curves of open and closed Ising chains with an odd number of spins exhibit an identical intermediate plateau, with only small quantitative differences appearing at moderate temperatures, which means that a geometric spin frustration of odd-membered rings is somewhat similar to the effect of open boundaries. The magnetization curves of the open Ising chains with an even number of spins differ drastically from those of the closed Ising chains due to the presence of an additional intermediate magnetization plateau. Furthermore, the initial susceptibility, inverse initial susceptibility, and susceptibility–temperature product are examined in detail as functions of temperature. These magnetic response functions demonstrate that the Curie constant and Weiss temperature represent fundamental characteristics of the magnetic system that are independent of the choice of boundary conditions.

## 1. Introduction

Exactly solved lattice-statistical models play an important role in the theoretical description of cooperative phenomena, because they provide rigorous insight into the relationship between microscopic interactions and macroscopic behavior [1,2,3,4]. Their pedagogical and scientific value is particularly significant as exact solutions often reveal fundamental physical mechanisms that may remain obscured in approximate treatments. Nevertheless, extracting physical insight from exactly solved interacting many-body systems is often challenging because corresponding exact solutions typically rely on sophisticated mathematical techniques. Among the most celebrated paradigmatic examples, one could mention the exact solution of the one-dimensional Ising model dating back more than a century ago in 1925 [5], and the exact solution of the two-dimensional Ising model reported by Onsager almost two decades later [6].

Over the past few decades, the Ising model has attained a unique status as one of the most influential and extensively studied lattice-statistical models with a remarkably diverse applicability across a wide range of disciplines, going far beyond its original motivation in magnetism [7,8,9,10]. Although the one-dimensional Ising model does not exhibit a genuine finite-temperature phase transition, in contrast to its two-dimensional counterpart [6], it still continues to attract considerable research interest. Recent studies have demonstrated the existence of pseudo-transitions in a certain class of one-dimensional Ising models that are reminiscent of true thermal phase transitions [11,12,13,14,15]. Moreover, one-dimensional Ising models provide valuable insight into a variety of intriguing phenomena, including the effect of long-range interaction [16,17], fractional magnetization plateaus in doped systems [18], and periodically modulated spin chains [19]. Consequently, the one-dimensional Ising model still remains an active and versatile research framework nearly a century after its original formulation.

Periodic boundary conditions are commonly assumed in most exact treatments of the Ising models, because they preserve translational invariance and considerably simplify the mathematical treatment [1,6]. Although the influence of boundary conditions vanishes in the thermodynamic limit, finite-size Ising spin systems may display remarkable differences depending on the choice of boundary conditions [7,8,20,21]. Consequently, the role of boundary conditions has attracted considerable attention in studies of finite-size effects and related phenomena in Ising spin systems [22,23,24,25,26]. From a methodological perspective, the transfer-matrix method represents one of the most powerful and widely used calculation techniques, enabling the exact treatment of one-dimensional Ising models [1,27,28,29]. In the conventional formulation, the transfer-matrix approach is most naturally implemented for periodic boundary conditions [1,2], while open boundary conditions are often considered to substantially complicate the transfer-matrix analysis [23,24,25,26]. Nevertheless, the transfer-matrix method can also be consistently adapted to finite Ising chains with open boundary conditions by explicitly accounting for boundary spins and individual matrix elements of the transfer matrix [20,21,22]. A systematic comparison between open and periodic boundary conditions is therefore particularly valuable for finite-size systems, where boundary effects may qualitatively modify magnetic and thermodynamic properties.

From an experimental perspective, recent advances in molecular magnetism have enabled the synthesis of a variety of finite spin systems with well-defined size and topology including molecular magnetic wheels, finite spin chains, and related low-dimensional magnetic clusters [30,31,32,33,34,35,36,37,38,39,40]. Such systems provide valuable experimental platforms for investigating finite-size effects that are absent in bulk magnetic materials. In particular, spin rings with an even number of magnetic centers cannot in principle undergo a geometric spin frustration [30,31,32,33,34,35], which may substantially modify the magnetic behavior of analogous odd-membered spin rings [36,37,38,39,40]. These observations motivate a systematic comparison between finite Ising chains with open and periodic boundary conditions, which allows one to discern the effect of open boundaries from those originating from the geometric spin frustration associated with odd-membered antiferromagnetic spin rings.

The present paper is organized as follows. Section 2 is devoted to the transfer-matrix treatment of the closed Ising chain with periodic boundary conditions, while Section 3 presents the corresponding transfer-matrix formulation for the open Ising chain with free boundary conditions. The obtained exact results for the thermodynamic quantities, magnetization process, parity effects, and magnetic response functions are discussed in Section 4. Finally, the main findings are summarized in Section 5.

## 2. Ising Chain Under the Periodic Boundary Condition

Let us consider first the spin-1/2 Ising chain with periodic boundary conditions given by the Hamiltonian

(1) $$H=J\sum_{i=1}^{N}\sigma_{i}\sigma_{i+1}-h\sum_{i=1}^{N}\sigma_{i},$$

where $\sigma_{i}=\pm \frac{1}{2}$ denotes the Ising spin located at the *i*th site of the one-dimensional chain, the first summation accounts for the nearest-neighbor exchange interaction, the second summation corresponds to the Zeeman energy associated with an external magnetic field *h*, and *N* denotes the total number of spins. For the *closed* Ising chain, it is necessary to impose the periodic boundary condition $\sigma_{N+1}\equiv \sigma_{1}$, which ensures the translational invariance of the system corresponding to an ideal crystal without boundaries.

The Hamiltonian (1) can be alternatively rewritten in the symmetric form

(2) $$H=\sum_{i=1}^{N}H_{i},$$

where

(3) $$H_{i}=J\sigma_{i}\sigma_{i+1}-\frac{h}{2}\left(\sigma_{i}+\sigma_{i+1}\right).$$

Using the symmetrized Hamiltonian (3), the partition function can be factorized into a product of terms involving only two adjacent spins

(4) $$\begin{array}{c} Z=\sum_{\sigma_{1}=\pm \frac{1}{2}}\sum_{\sigma_{2}=\pm \frac{1}{2}}\ldots \sum_{\sigma_{N}=\pm \frac{1}{2}}\prod_{i=1}^{N}\exp\left[-\beta J\sigma_{i}\sigma_{i+1}+\frac{\beta h}{2}\left(\sigma_{i}+\sigma_{i+1}\right)\right]. \end{array}$$

Each factor in the product (4) can be formally replaced by the expression $\langle \sigma_{i}\left|T\right|\sigma_{i+1}\rangle$ defined as

(5) $$\begin{array}{c} \langle \sigma_{i}\left|T\right|\sigma_{i+1}\rangle =\exp\left[-\beta J\sigma_{i}\sigma_{i+1}+\frac{\beta h}{2}\left(\sigma_{i}+\sigma_{i+1}\right)\right], \end{array}$$

which depends only on two nearest-neighboring spins $\sigma_{i}$ and $\sigma_{i+1}$. The expression (5) may alternatively be interpreted as 2×2 matrix once all possible spin-state values ($\sigma_{i},\sigma_{i+1}=\pm 1/2$) are considered with the first spin $\sigma_{i}$ defining its rows and the second spin $\sigma_{i+1}$ defining its columns

(6) $$\begin{array}{c} \langle \sigma_{i}\left|T\right|\sigma_{i+1}\rangle =\left(\begin{array}{cc} \langle +\left|T\right|+\rangle & \langle +\left|T\right|-\rangle \\ \langle -\left|T\right|+\rangle & \langle -\left|T\right|-\rangle \end{array}\right)=\left(\begin{array}{cc} \exp\left(-\frac{\beta J}{4}+\frac{\beta h}{2}\right) & \exp\left(\frac{\beta J}{4}\right)\\ \exp\left(\frac{\beta J}{4}\right) & \exp\left(-\frac{\beta J}{4}-\frac{\beta h}{2}\right) \end{array}\right), \end{array}$$

which is commonly referred to as the transfer matrix [1]. Introducing the transfer matrix (5) and (6) allows factorization of the partition function

(7) $$\begin{array}{c} Z=\sum_{\sigma_{1}=\pm \frac{1}{2}}\sum_{\sigma_{2}=\pm \frac{1}{2}}\ldots \sum_{\sigma_{N}=\pm \frac{1}{2}}\prod_{i=1}^{N}\langle \sigma_{i}\left|T\right|\sigma_{i+1}\rangle . \end{array}$$

When adapting the transfer-matrix formalism, the summations over the spin states $\sigma_{2},\sigma_{3},\ldots ,\sigma_{N}$ can be interpreted as successive matrix multiplications yielding

(8) $$\begin{array}{ccc} Z\;\; & = & \;\;\sum_{\sigma_{1}=\pm \frac{1}{2}}\sum_{\sigma_{2}=\pm \frac{1}{2}}\ldots \sum_{\sigma_{N}=\pm \frac{1}{2}}\langle \sigma_{1}\left|T\right|\sigma_{2}\rangle \langle \sigma_{2}\left|T\right|\sigma_{3}\rangle \ldots \langle \sigma_{i}\left|T\right|\sigma_{i+1}\rangle \ldots \langle \sigma_{N}\left|T\right|\sigma_{1}\rangle \\ \;\;\;\; & = & \;\;\;\;\sum_{\sigma_{1}=\pm \frac{1}{2}}\sum_{\sigma_{3}=\pm \frac{1}{2}}\ldots \sum_{\sigma_{N}=\pm \frac{1}{2}}\langle \sigma_{1}\left|T^{2}\right|\sigma_{3}\rangle \ldots \langle \sigma_{i}\left|T\right|\sigma_{i+1}\rangle \ldots \langle \sigma_{N}\left|T\right|\sigma_{1}\rangle \\ \;\;\;\; & = & \;\;\;\;\sum_{\sigma_{1}=\pm \frac{1}{2}}\langle \sigma_{1}\left|T^{N}\right|\sigma_{1}\rangle =\langle +\left|T^{N}\right|+\rangle +\langle -\left|T^{N}\right|-\rangle =\mathrm{Tr}\;T^{N}. \end{array}$$

The problem of evaluating the partition function of the closed Ising chain thus reduces to calculating the trace of the *N*th power of the transfer matrix. To proceed further, one may apply a unitary transformation that diagonalizes the transfer matrix *T*, i.e., $U^{-1}TU=\Lambda$, where Λ denotes a diagonal matrix. Since the trace is invariant under cyclic permutations of matrices, the partition function can be expressed in terms of the transfer-matrix eigenvalues $\lambda_{\pm }$

(9) $$\begin{array}{c} Z=\mathrm{Tr}\;T^{N}=\mathrm{Tr}\;\left(UU^{-1}TUU^{-1}TU\ldots U^{-1}T\right)=\mathrm{Tr}\;\Lambda^{N}=\lambda_{+}^{N}+\lambda_{-}^{N}. \end{array}$$

The periodic boundary condition implies that the partition function of the closed Ising chain reduces to a simple sum of the *N*th powers of the transfer-matrix eigenvalues.

The eigenvalues corresponding to the transfer-matrix (6) can be determined from the characteristic equation

(10) $$\begin{array}{c} \left|\begin{array}{cc} \exp\left(-\frac{\beta J}{4}+\frac{\beta h}{2}\right)-\lambda_{i} & \exp\left(\frac{\beta J}{4}\right)\\ \exp\left(\frac{\beta J}{4}\right) & \exp\left(-\frac{\beta J}{4}-\frac{\beta h}{2}\right)-\lambda_{i} \end{array}\right|=0, \end{array}$$

which yields the following two roots

(11) $$\begin{array}{c} \lambda_{\pm }=\exp\left(-\frac{\beta J}{4}\right)\left[\cosh\left(\frac{\beta h}{2}\right)\pm \sqrt{\sinh^{2}\left(\frac{\beta h}{2}\right)+\exp\left(\beta J\right)}\right]. \end{array}$$

Having determined the transfer-matrix eigenvalues, one may calculate for the closed Ising chain all magnetic and thermodynamic quantities of interest. The Gibbs free energy per spin is given by

(12) $$\begin{array}{c} g=-\frac{1}{N}k_{B}T\ln Z=-\frac{1}{N}k_{B}T\ln\left(\lambda_{+}^{N}+\lambda_{-}^{N}\right). \end{array}$$

The isothermal magnetization of the closed Ising chain is then obtained from the relation

(13) $$m=-{\left(\frac{𝜕g}{𝜕h}\right)}_{T}=\frac{1}{2}\frac{\sinh\left(\frac{\beta h}{2}\right)}{\sqrt{\sinh^{2}\left(\frac{\beta h}{2}\right)+\exp\left(\beta J\right)}}\frac{\lambda_{+}^{N}-\lambda_{-}^{N}}{\lambda_{+}^{N}+\lambda_{-}^{N}},$$

which reduces in the thermodynamic limit N→∞ to the well-known formula

(14) $$m=\frac{1}{2}\frac{\sinh\left(\frac{\beta h}{2}\right)}{\sqrt{\sinh^{2}\left(\frac{\beta h}{2}\right)+\exp\left(\beta J\right)}}.$$

The isothermal initial susceptibility is subsequently determined from the relation

(15) $$\chi ={\left(\frac{𝜕m}{𝜕h}\right)}_{T,h=0}=\frac{C}{T}\exp\left(-\frac{\beta J}{2}\right)\frac{1-{\left(-1\right)}^{N}\tanh^{N}\left(\frac{\beta J}{4}\right)}{1+{\left(-1\right)}^{N}\tanh^{N}\left(\frac{\beta J}{4}\right)},$$

where $C=\frac{1}{4}$ denotes the Curie constant. In the high-temperature limit, the latter expression reduces to the familiar Curie–Weiss form

(16) $$\chi =\frac{C}{T-\Theta },$$

where $\Theta =-\frac{J}{2k_{B}}$ denotes the Weiss constant.

## 3. Ising Chain Under the Open Boundary Condition

Let us now adapt the transfer-matrix approach to the open Ising chain with free boundary conditions. The Hamiltonian of the spin-1/2 Ising chain under the open boundary conditions reads

(17) $$H=J\sum_{i=1}^{N-1}\sigma_{i}\sigma_{i+1}-h\sum_{i=1}^{N}\sigma_{i}.$$

The only difference between the Hamiltonians (1) and (17) of the closed and open Ising chains is the absence of one interaction term, which ensures the translational invariance of the closed Ising chain. By contrast, the open Ising chain is considered under free boundary conditions, where the first and last Ising spins $\sigma_{1}$ and $\sigma_{N}$ represent the boundary spins of a finite chain interacting with only one neighboring spin, unlike all other spins from the bulk.

Most aspects of the calculation procedure remain unchanged. The Hamiltonian (17) of the open Ising chain can be rewritten into the symmetrized form

(18) $$H=\sum_{i=1}^{N-1}\left[J\sigma_{i}\sigma_{i+1}-\frac{h}{2}\left(\sigma_{i}+\sigma_{i+1}\right)\right]-\frac{h}{2}\left(\sigma_{1}+\sigma_{N}\right),$$

where the summation extends over one less bond, but two additional terms associated with the magnetic field acting on the first and last spins appear instead. It should be stressed, however, that the definition of the transfer matrix remains unchanged [see Equations (5) and (6)]. The partition function can still be factorized into the product

(19) $$\begin{array}{c} Z=\sum_{\sigma_{1}=\pm \frac{1}{2}}\sum_{\sigma_{2}=\pm \frac{1}{2}}\;\;\ldots \;\;\sum_{\sigma_{N}=\pm \frac{1}{2}}\;\;\;\exp\left[\frac{\beta h}{2}\left(\sigma_{1}\;+\;\sigma_{N}\right)\right]\left\{\prod_{i=1}^{N-1}\;\exp\left[-\beta J\sigma_{i}\sigma_{i+1}\;+\;\frac{\beta h}{2}\left(\sigma_{i}\;+\;\sigma_{i+1}\right)\right]\;\right\}, \end{array}$$

where the expressions in curly brackets can be identified as a product of the transfer matrices (6)

(20) $$\begin{array}{ccc} Z\;\;\; & = & \;\;\;\sum_{\sigma_{1}=\pm \frac{1}{2}}\sum_{\sigma_{2}=\pm \frac{1}{2}}\ldots \;\;\;\sum_{\sigma_{N}=\pm \frac{1}{2}}\exp\left[\frac{\beta h}{2}\left(\sigma_{1}+\sigma_{N}\right)\right]\langle \sigma_{1}\left|T\right|\sigma_{2}\rangle \langle \sigma_{2}\left|T\right|\sigma_{3}\rangle \ldots \langle \sigma_{N-1}\left|T\right|\sigma_{N}\rangle \\ \;\;\; & = & \;\;\;\sum_{\sigma_{1}=\pm \frac{1}{2}}\sum_{\sigma_{N}=\pm \frac{1}{2}}\exp\left[\frac{\beta h}{2}\left(\sigma_{1}+\sigma_{N}\right)\right]\langle \sigma_{1}\left|T^{N-1}\right|\sigma_{N}\rangle . \end{array}$$

Under the open boundary conditions, the successive summation over the states of the bulk spins $\sigma_{2},\sigma_{3},\ldots ,\sigma_{N-1}$ yields an expression depending on both boundary spins $\sigma_{1}$ and $\sigma_{N}$. Consequently, the partition function of the open Ising chain can be expressed in terms of all four elements of the (N−1)th power of the transfer matrix (6).

To proceed further, we will employ the spectral decomposition of the transfer matrix (6) using the eigenvectors, which correspond to two already reported eigenvalues (11) to be reindexed as $\lambda_{1,2}$ instead of $\lambda_{+,-}$. The corresponding eigenvectors $|\varphi_{1}\rangle$ and $|\varphi_{2}\rangle$ of the transfer matrix (6) follow from solving the eigenvalue problem

(21) $$T|\varphi_{j}\rangle =\lambda_{j}|\varphi_{j}\rangle ;\;|\varphi_{j}\rangle =a_{j}|+\rangle +b_{j}|-\rangle ;\;j=1,2.$$

Substituting the transfer matrix (6) into Equation (21) leads to a set of two linearly dependent equations for the coefficients $a_{j}$ and $b_{j}$

(22) $$\left(\begin{array}{cc} \exp\left(-\frac{\beta J}{4}+\frac{\beta h}{2}\right)-\lambda_{j} & \exp(\frac{\beta J}{4})\\ \exp(\frac{\beta J}{4}) & \exp\left(-\frac{\beta J}{4}-\frac{\beta h}{2}\right)-\lambda_{j} \end{array}\right)\left(\begin{array}{c} a_{j}\\ b_{j} \end{array}\right)=0$$

from which the coefficients $a_{j}$ and $b_{j}$ can be expressed in terms of each other as

(23) $$a_{j}=b_{j}\frac{\exp(\frac{\beta J}{4})}{\lambda_{j}-\exp\left(-\frac{\beta J}{4}+\frac{\beta h}{2}\right)},\;a_{j}=b_{j}\frac{\lambda_{j}-\exp\left(-\frac{\beta J}{4}-\frac{\beta h}{2}\right)}{\exp(\frac{\beta J}{4})}.$$

Using the normalization condition $a_{j}^{2}+b_{j}^{2}=1$ for the eigenvectors, the coefficients $a_{j}$ and $b_{j}$ determining both eigenvectors are given by

(24) $$a_{1}=a,\;b_{1}=b,\;a_{2}=b,\;b_{2}=-a,$$

where

(25) $$a=\frac{1}{\sqrt{2}}\sqrt{1+\frac{\sinh(\frac{\beta h}{2})}{\sqrt{\sinh^{2}\left(\frac{\beta h}{2}\right)+\exp\left(\beta J\right)}}};\;b=\frac{1}{\sqrt{2}}\sqrt{1-\frac{\sinh(\frac{\beta h}{2})}{\sqrt{\sinh^{2}\left(\frac{\beta h}{2}\right)+\exp\left(\beta J\right)}}}.$$

The spectral decomposition can now be employed to determine the (N−1)th power of the transfer matrix

(26) $$T^{N-1}=T^{N-1}\sum_{i=1}^{2}|\varphi_{j}\rangle \langle \varphi_{j}|=\sum_{j=1}^{2}T^{N-1}|\varphi_{j}\rangle \langle \varphi_{j}|=\sum_{j=1}^{2}\lambda_{j}^{N-1}|\varphi_{j}\rangle \langle \varphi_{j}|.$$

All four transfer-matrix elements $\langle \sigma_{1}|T^{N-1}|\sigma_{N}\rangle$ can be obtained from Equation (26) by taking scalar products with the basis state vectors

(27) $$\begin{array}{cc} \;\; & \;\;\langle +|T^{N-1}|+\rangle =\sum_{j=1}^{2}\lambda_{j}^{N-1}\langle +|\varphi_{j}\rangle \langle \varphi_{j}|+\rangle =a^{2}\lambda_{1}^{N-1}+b^{2}\lambda_{2}^{N-1},\\ \;\; & \;\;\langle +|T^{N-1}|-\rangle =\sum_{j=1}^{2}\lambda_{j}^{N-1}\langle +|\varphi_{j}\rangle \langle \varphi_{j}|-\rangle =ab\lambda_{1}^{N-1}-ab\lambda_{2}^{N-1},\\ \;\; & \;\;\langle -|T^{N-1}|+\rangle =\sum_{j=1}^{2}\lambda_{j}^{N-1}\langle -|\varphi_{j}\rangle \langle \varphi_{j}|+\rangle =ab\lambda_{1}^{N-1}-ab\lambda_{2}^{N-1},\\ \;\; & \;\;\langle -|T^{N-1}|-\rangle =\sum_{j=1}^{2}\lambda_{j}^{N-1}\langle -|\varphi_{j}\rangle \langle \varphi_{j}|-\rangle =b^{2}\lambda_{1}^{N-1}+a^{2}\lambda_{2}^{N-1}\;\;. \end{array}$$

Substituting the individual matrix elements into Equation (20) finally yields the partition function of the open Ising chain in the form

$$\begin{array}{ccc} Z\;\;\; & = & \;\;\;\sum_{\sigma_{1}=\pm \frac{1}{2}}\sum_{\sigma_{2}=\pm \frac{1}{2}}\exp\left[\frac{\beta h}{2}\left(\sigma_{1}+\sigma_{N}\right)\right]\langle \sigma_{1}|T^{N-1}|\sigma_{N}\rangle \\ \;\;\; & = & \;\;\;\exp\left(\frac{\beta h}{2}\right)\langle +|T^{N-1}|+\rangle +\exp\left(-\frac{\beta h}{2}\right)\langle -|T^{N-1}\left|-\rangle +\langle +\right|T^{N-1}\left|-\rangle +\langle -\right|T^{N-1}|+\rangle \\ \;\;\; & = & \;\;\;\left[\exp\left(\;\frac{\beta h}{2}\;\right)a^{2}\;+\;\exp\left(\;-\frac{\beta h}{2}\;\right)b^{2}\;+\;2ab\right]\lambda_{1}^{N-1}\;+\;\left[\exp\left(\;\frac{\beta h}{2}\;\right)b^{2}\;+\;\exp\left(\;-\frac{\beta h}{2}\;\right)a^{2}\;-\;2ab\right]\lambda_{2}^{N-1}. \end{array}$$

Having derived the partition function, all relevant magnetic and thermodynamic quantities for the open Ising chain can be calculated straightforwardly. In particular, the Gibbs free energy per spin is given by

(28) $$\begin{array}{ccc} g\; & = & \;-\frac{1}{N}k_{B}T\ln\left\{\left[\exp\left(\frac{\beta h}{2}\right)a^{2}\;+\;\exp\left(-\frac{\beta h}{2}\right)b^{2}\;+\;2ab\right]\lambda_{1}^{N-1}\right.\\ & + & \left.\left[\exp\left(\frac{\beta h}{2}\right)b^{2}\;+\;\exp\left(-\frac{\beta h}{2}\right)a^{2}\;-\;2ab\right]\lambda_{2}^{N-1}\right\}, \end{array}$$

while the magnetization can be obtained from Equation (28) using the standard relation m=−𝜕g/𝜕h, but its final formula is too cumbersome to write it down here explicitly. The isothermal initial susceptibility follows from the relation

(29) $$\begin{array}{ccc} \chi \; & = & \;{\left(\frac{𝜕m}{𝜕h}\right)}_{T,h=0}=\frac{C}{T}\exp\left(-\frac{\beta J}{2}\right)+\frac{\beta }{2}\exp\left(-\frac{\beta J}{4}\right)\sinh\left(\frac{\beta J}{4}\right)\\ & & \left\{1+\exp\left(-\frac{\beta J}{4}\right)\sinh\left(\frac{\beta J}{4}\right)\left[{\left(-1\right)}^{N}\tanh^{N-1}\left(\frac{\beta J}{4}\right)-1\right]\right\}. \end{array}$$

## 4. Results and Discussion

**Figure 1:**
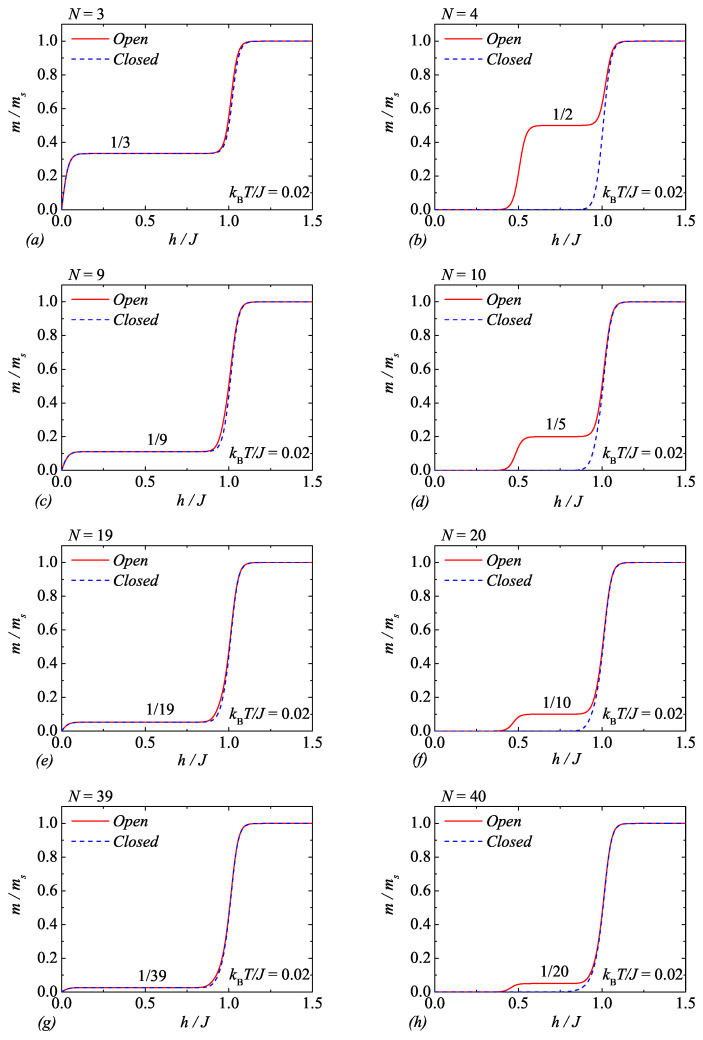
Low-temperature magnetization curves of antiferromagnetic Ising chains with open and periodic boundary conditions for several chain lengths: (**a**) N=3, (**b**) N=4, (**c**) N=9, (**d**) N=10, (**e**) N=19, (**f**) N=20, (**g**) N=39, (**h**) N=40. The left (right) panels display the magnetization curves for chains with an odd (even) number of spins.

We first examine the low-temperature magnetization process of antiferromagnetic Ising chains with open and periodic boundary conditions, which are depicted in Figure 1 for several chain lengths. As evidenced by the left panels of Figure 1, the magnetization curves of open and closed Ising chains with an odd number of spins (N=3, 9, 19, and 39) exhibit qualitatively similar behavior at low temperatures. In particular, both open and closed chains display a single intermediate magnetization plateau at 1/N of the saturation magnetization, although its microscopic origin differs substantially. While the intermediate plateau of the open Ising chains with an odd number of spins originates from the magnetization of the boundary spins, the same intermediate plateau of the closed Ising chains results from a geometric spin frustration inherent to odd-membered spin rings. As expected, the differences between open and closed chains become progressively less pronounced with increasing chain length.

By contrast, the magnetization curves of open and closed Ising chains with an even number of spins (N=4, 10, 20, and 40) illustrated in the right panels of Figure 1 differ qualitatively. While the closed Ising chains display only a zero-magnetization plateau followed by a direct field-driven transition to the fully polarized state, the open Ising chains exhibit an additional intermediate plateau at 2/N of the saturation magnetization. This distinction can be understood from the corresponding ground-state spin configurations. For the closed Ising chains with an even number of spins, the low-field ground state is the perfect antiferromagnetic Néel order with alternating ‘up’ and ‘down’ spins, yielding a zero-magnetization plateau. This antiferromagnetic state remains stable up to the saturation field, at which all spins oriented opposite to the magnetic field reverse simultaneously in the direction of the magnetic-field. The open Ising chains contrarily exhibit an intermediate magnetization plateau at 2/N of the saturation magnetization, because the boundary spin aligned antiparallel to the magnetic field undergoes a spin reversal at half the field required to flip the bulk spins. Consequently, the magnetization increases in a two-step process, giving rise to the additional plateau at 2/N of the saturation magnetization.

**Figure 2:**
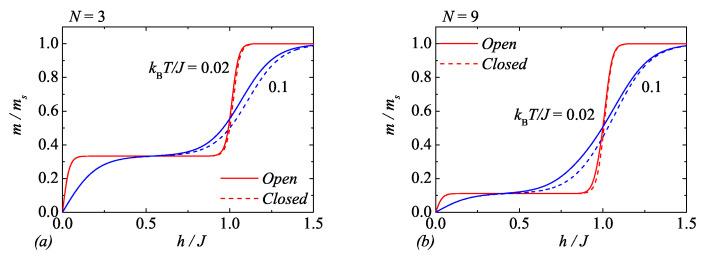
Magnetization curves of open and closed antiferromagnetic Ising chains with N=3 (**a**) and N=9 (**b**) spins as a function of the external magnetic field at two different temperatures.

The results discussed above might suggest that the distinction between open and closed Ising chains is largely irrelevant for chains with an odd number of spins, whereas the boundary conditions play a crucial role for chains with an even number of spins. However, this conclusion is valid only at sufficiently low temperatures such as $k_{B}T/J\approx 0.02$. To illustrate this point, Figure 2 compares the isothermal magnetization curves of open and closed Ising chains with an odd number of spins N=3 and N=9 at low and moderate temperatures $k_{B}T/J=0.02$ and 0.1, respectively. It is evident from this figure that the quantitative differences in the magnetization curves become more pronounced at the moderate temperature $k_{B}T/J=0.1$. On the other hand, the differences in the isothermal magnetization curves diminish again upon further increasing the temperature, although the high-temperature magnetization curves are not shown in Figure 2 for the sake of clarity.

**Figure 3:**
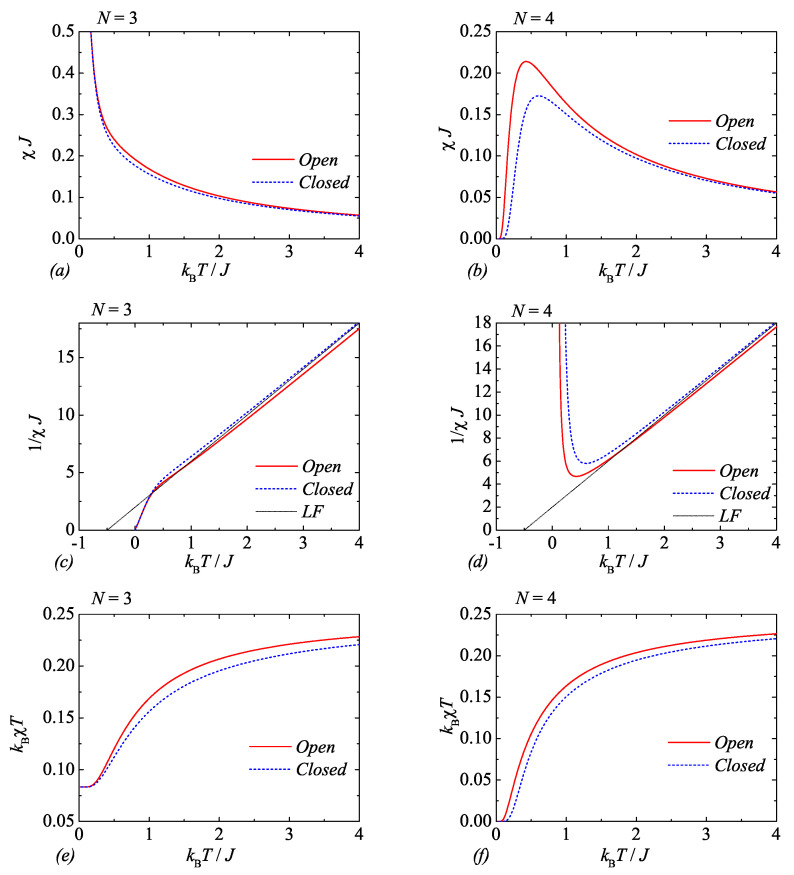
Temperature dependences of the initial susceptibility for N=3 (**a**) and N=4 (**b**), the inverse susceptibility for N=3 (**c**) and N=4 (**d**), and the susceptibility times temperature product for N=3 (**e**) and (**f**) for open and closed Ising chains.

Next, we examine the influence of the chain length and boundary conditions on the initial susceptibility of the open and closed Ising chains. We begin our discussion by considering the shortest chains with odd and even numbers of spins. The temperature dependence of the initial susceptibility for the open and closed Ising chains, with the odd number of spins N=3, is depicted in Figure 3a. In both cases, the susceptibility diverges as the temperature approaches zero due to the ground state with a nonzero total spin, which necessarily arises from the odd number of spins. The susceptibility of the open and closed chains decreases with increasing temperature, although the decrease is noticeably more gradual for the open chain than for the closed one. For comparison, the temperature dependence of the initial susceptibility of open and closed Ising chains with an even number of spins is shown in Figure 3b for N=4. In contrast to the odd-spin case, the susceptibility exhibits the characteristic behavior of an antiferromagnetic system with a fully compensated ground state having zero total spin moment. Consequently, the susceptibility initially starts from zero until it reaches a pronounced round maximum and then gradually decreases at higher temperatures. Similar to the case with an odd number of spins, the initial susceptibility of the open chain remains slightly higher than that of the corresponding closed chain over the entire temperature range. The temperature dependence of the inverse initial susceptibility of the open and closed Ising chains with N=3 is shown in Figure 3c. The inverse susceptibility monotonically increases with temperature, with an initial steeper low-temperature rise followed by a less steep linear dependence, which is characteristic of the paramagnetic regime $k_{B}T/J\to \infty$. This linear dependence is represented by the linear-fit (LF) curve accurately reproducing the high-temperature behavior of both open and closed Ising chains, whereby its intersection with the temperature axis yields the Weiss constant $\Theta =-\frac{1}{2}$ in agreement with the Curie–Weiss law $\frac{1}{\chi }=\frac{T}{C}-\frac{\Theta }{C}$. For the open and closed chains with an even number of spins N=4, the inverse susceptibility decreases upon lowering the temperature until it reaches a local minimum before eventually diverging as the temperature approaches zero; see Figure 3d. Despite these qualitative differences, the linear-fit (LF) curves shown in Figure 3 indicate the Weiss constant is independent of both chain length and the choice of boundary conditions. Last but not least, the temperature dependence of the initial susceptibility times temperature product χT for the open and closed Ising chains with N=3 is shown in Figure 3e. The effective magnetic moment represented by the quantity χT attains a finite zero-temperature value 1/12 equal to the ratio C/N between the Curie constant and the number of spins, and subsequently increases monotonically with temperature toward the asymptotic value 1/4 corresponding to the Curie constant. On the other hand, the temperature dependence of the susceptibility times temperature product for the open and closed Ising chains with N=4 is depicted in Figure 3f. For the open and closed chains consisting of an even number of spins, the effective magnetic moment $k_{B}\chi T$ vanishes at zero temperature and subsequently increases monotonically toward the Curie constant with increasing temperature regardless of the boundary conditions.

**Figure 4:**
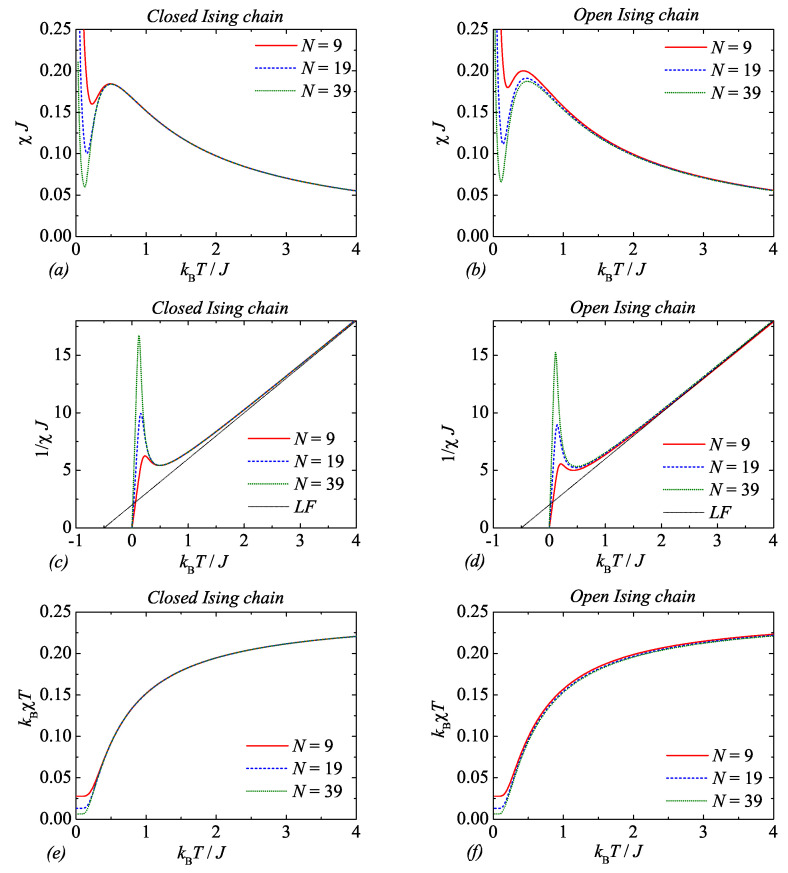
Temperature dependences of the initial susceptibility (**a**,**b**), inverse susceptibility (**c**,**d**), and susceptibility times temperature product (**e**,**f**) for closed Ising chains (**a**,**c**,**e**) and open Ising chains (**b**,**d**,**f**) with several chain lengths N=9, 19, and 39.

To gain deeper insight into the effect of system size on the magnetic response function of the Ising chains under different boundary conditions, Figure 4 presents the initial susceptibility, inverse susceptibility, and susceptibility times temperature product for the closed (left panels) and open (right panels) chains with a few different values of the odd number of spins N=9, 19, and 39. The influence of the chain length is most pronounced at low temperatures, whereas the differences between individual system sizes gradually vanish with increasing temperatures. Moreover, the finite-size effects are qualitatively similar for both boundary conditions, since the same thermodynamic behavior must ultimately be recovered in the thermodynamic limit N→∞. The magnetic susceptibility of the closed and open Ising chains with the odd number of spins shown in Figure 4a,b initially increases upon lowering the temperature to reach a local maximum, then decreases to a local minimum, and finally diverges in the low-temperature regime. This behavior reflects the competition between antiferromagnetic correlations suppressing the susceptibility at intermediate temperatures and the paramagnetic contribution of a single uncompensated spin dominating at sufficiently low temperatures. As the chain length increases, the local minimum becomes progressively more pronounced because the relative contribution of a single uncompensated spin decreases as 1/N. The corresponding inverse susceptibility of the closed and open Ising chains with the odd number of spins shown in Figure 4c,d naturally exhibits complementary behavior: it first decreases upon cooling to a local minimum, then exhibits a pronounced peak before completely vanishing as the temperature tends to zero. The observed round maximum becomes progressively higher and sharper with increasing system size, reflecting the growing separation between the antiferromagnetic and paramagnetic regimes. In agreement with the Curie–Weiss law, the linear-fit (LF) curve constructed in the high-temperature region extrapolates to the common intercept −1/2, confirming that the Weiss temperature is independent of both the system size and the choice of boundary conditions. The temperature dependence of the initial susceptibility times temperature product for the closed and open Ising chains with the odd number of spins N=9, 19, and 39 are shown in Figure 4e,f. The effective magnetic moment χT again approaches a finite zero-temperature value equal to the ratio C/N between the Curie constant and the number of spins owing to the paramagnetic contribution of a single uncompensated spin. With increasing temperature, the quantity χT rises steadily and gradually converges to the asymptotic value consistent with the Curie constant $\frac{1}{4}$.

**Figure 5:**
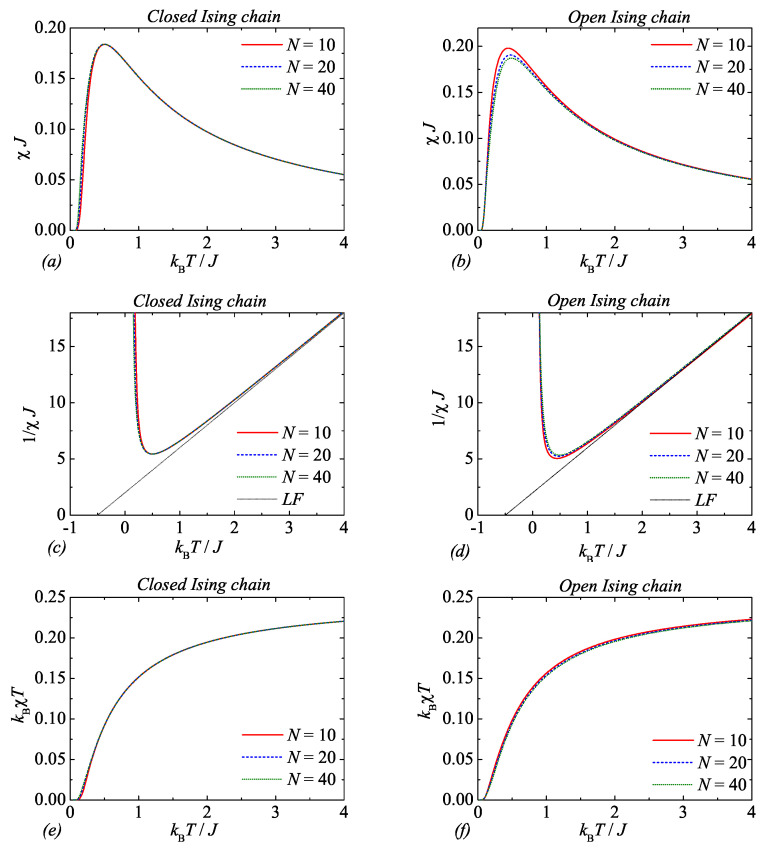
Temperature dependences of the initial susceptibility (**a**,**b**), inverse susceptibility (**c**,**d**), and susceptibility times temperature product (**e**,**f**) for closed (**a**,**c**,**e**) and open (**b**,**d**,**f**) Ising chains with several chain lengths N=10, 20, and 40.

Finally, we discuss typical temperature variations in the magnetic response functions (the initial susceptibility, inverse susceptibility, and susceptibility times temperature product) of the closed and open Ising chains with an even number of spins, as depicted in Figure 5 for N=10, 20, and 40. All three quantities exhibit the same qualitative behavior as that already discussed for the shortest chain, with the even number of spins N=4 shown in the right panels of Figure 3. Therefore, the following discussion focuses primarily on a detailed analysis of finite-size effects. The influence of the chain length is markedly different for closed and open Ising chains. In closed chains, the magnetic response functions exhibit only relatively small finite-size corrections, which are most apparent at very low temperatures. By contrast, the finite-size effects are considerably more pronounced in open chains. In both cases, however, the differences between individual chain lengths diminish with the increasing system size, as evidenced by the substantially smaller deviations between the results for N=20 and N=40 compared with those between N=10 and N=20. This behavior is consistent with the convergence toward the common thermodynamic limit. A notable distinction between the open and periodic boundary conditions concerns the temperature range where finite-size effects are most significant. For closed chains, the influence of the chain length is largely confined to the low-temperature region. On the other hand, open chains display the most pronounced finite-size effects at intermediate temperatures around $k_{B}T/J\approx 0.5$, where the contribution of the boundary spins to the magnetic response functions remains appreciable.

## 5. Conclusions

In the present work, the transfer-matrix method has been adapted to obtain exact results for the spin-1/2 Ising chain under open boundary conditions, which were systematically compared with the corresponding exact results for the same model under periodic boundary conditions obtained within a more common formulation of the transfer-matrix method. Exact analytical expressions for the partition functions, magnetization, and magnetic susceptibility were derived for both open and closed Ising chains with an arbitrary number of spins. These exact results enabled a comprehensive analysis of the effects of boundary conditions, chain length, and parity on the magnetic properties of finite antiferromagnetic Ising chains.

Particular attention was devoted to the role of parity in determining the magnetic behavior of antiferromagnetic Ising chains in an external magnetic field. It was shown that the open and closed Ising chains with an odd number of spins exhibit qualitatively similar magnetization curves, including an identical intermediate magnetization plateau, despite their fundamentally different microscopic origin. While the intermediate plateau of the closed chains arises from a geometric spin frustration associated with the odd number of spins and periodic boundary conditions, the same magnetization plateau of the open chains originates from the boundary effects since the boundary spins are magnetized at half the magnetic field required for magnetizing the bulk spins. The intermediate plateau related to the magnetization of the boundary spins is also present in the open Ising chains with an even number of spins, whereas it is naturally absent in the closed Ising chains with an even number of spins, that displays only a zero-magnetization plateau arising from a perfect antiferromagnetic spin arrangement instead.

The presented results thus provide compelling evidence that boundary effects can substantially modify the low-temperature features of finite-size Ising chains (including the presence or absence of an intermediate magnetization plateau), although their high-temperature features, reflected predominantly through the Curie and Weiss constants, naturally remain independent of the choice of boundary conditions. The present study therefore provides a clear and pedagogically transparent illustration of how boundary conditions influence the magnetic properties of finite-size Ising spin systems within the framework of statistical mechanics. Beyond its conceptual relevance, the transfer-matrix formulation developed for the open Ising chains may serve as a useful starting point for future investigations of more complex finite-size spin systems. Possible extensions include mixed-spin Ising chains and ladders, low-dimensional decorated Ising spin systems, or even more complex Ising-Heisenberg spin systems, for which boundary effects and finite-size phenomena may give rise to similarly rich magnetic behavior.

## Data Availability

The original contributions presented in this study are included in the article in the form of presented exact analytical formulas, from which all data sets presented in the figures can be obtained. Further inquiries can be directed to the corresponding author.

## Abbreviations

The following abbreviations are used in this manuscript:

OBC	Open boundary conditions
PBC	Periodic boundary conditions
LF	Linear fit
